# The UK AVATAR 1 and 2 Trials for People with Distressing Voices – Findings and Learning from AVATAR1, and AVATAR2 Developments in Theory and Therapy

**DOI:** 10.1192/j.eurpsy.2022.84

**Published:** 2022-09-01

**Authors:** T. Craig, P. Garety, T. Ward, C. Edwards, M. Rus-Calafell, M. Huckvale, R. Emsley

**Affiliations:** 1 Kings College London, Institute of Psychiatry, Psychology and Neuroscience, Department Of Health Service And Population, London, United Kingdom; 2 King’s College London, Psychology, London, United Kingdom; 3 Ruhr-Universitat Bochum, Psychology, Bochum, Germany; 4 University College London, Speech, Hearing And Phonetic Sciences, London, United Kingdom; 5 King’s College London, Biostatistics, London, United Kingdom

**Keywords:** Auditory Verbal Hallucinations, Psychological therapy, Psychosis, clinical trial

## Abstract

**Introduction:**

Many people suffering from psychotic disorders report persistent auditory verbal hallucinations (‘voices’) despite pharmacological and psychological therapy. Interest is growing in approaches that emphasise the personal relationship between the patient and their voice(s). AVATAR therapy is one such approach that uses a digital representation (avatar) of a selected voice to facilitate a three-way discussion between patient, therapist and voice, the therapist speaking either as him/herself or in the digitally transformed voice of the avatar. Objectives: To describe AVATAR therapy and an ongoing multi-centre clinical trial. Methods: Encouraging findings from an earlier controlled trial (AVATAR1) comparing AVATAR therapy and supportive counselling informed our current multi-site cost-effectiveness trial of brief and extended versions of the therapy compared to treatment as usual (AVATAR2). Results: AVATAR1 delivered in 7 weekly sessions resulted in a reduction in the frequency, distress and power of voices that was significantly superior to supportive counselling. Clinical experience suggested that some participants improved in response to the early focus on anxiety while others seemed more responsive to later more formulation-driven approach. These findings led us to the current ongoing three arm clinical trial comprising a brief (6 session) focus on anxiety/assertiveness, an extended (12 session) formulation-driven approach both approaches compared to treatment as usual. Conclusion: Previous AVATAR studies suggest this is a therapy with considerable promise. It can be delivered through widely available laptop computers, usually in clinic but also remotely via existing commercial platforms. The current trial will address questions about dissemination, training and cost-effectiveness in NHS settings.

**Disclosure:**

The digital technology employed in AVATAR therapy is provided by licence for the trial from Avatar Therapy Ltd

